# Effect of Thermal Shock Conditions on the Low-Cycle Fatigue Performance of 3D-Printed Materials: Acrylonitrile Butadiene Styrene, Acrylonitrile-Styrene-Acrylate, High-Impact Polystyrene, and Poly(lactic acid)

**DOI:** 10.3390/polym16131823

**Published:** 2024-06-27

**Authors:** Marcin Głowacki, Adam Mazurkiewicz, Katarzyna Skórczewska, Krzysztof Lewandowski, Emil Smyk, Ricardo Branco

**Affiliations:** 1 Faculty of Mechanical Engineering, Bydgoszcz University of Science and Technology, Kaliskiego 7 Street, 85-796 Bydgoszcz, Poland; adam.mazurkiewicz@pbs.edu.pl (A.M.); emil.smyk@pbs.edu.pl (E.S.); 2 Faculty of Chemical Technology and Engineering, Bydgoszcz University of Science and Technology, Seminaryjna 3 Street, 85-326 Bydgoszcz, Poland; katarzyna.skorczewska@pbs.edu.pl (K.S.); krzysztof.lewandowski@pbs.edu.pl (K.L.); 3 ARISE, CEMMPRE, Department of Mechanical Engineering, University of Coimbra, 3030-788 Coimbra, Portugal; ricardo.branco@dem.uc.pt

**Keywords:** polymers, 3D print, fatigue life, cyclic test, thermal shock, microCT

## Abstract

3D printing technology is becoming a widely adopted alternative to traditional polymer manufacturing methods. The most important advantage of 3D printing over traditional manufacturing methods, such as injection molding or extrusion, is the short time from the creation of a new design to the finished product. Nevertheless, 3D-printed parts generally have lower strength and lower durability compared to the same parts manufactured using traditional methods. Resistance to the environmental conditions in which a 3D-printed part operates is important to its durability. One of the most important factors that reduces durability and degrades the mechanical properties of 3D-printed parts is temperature, especially rapid temperature changes. In the case of inhomogeneous internal geometry and heterogeneous material properties, rapid temperature changes can have a significant impact on the degradation of 3D-printed parts. This degradation is more severe in high-humidity environments. Under these complex service conditions, information on the strength and fatigue behavior of 3D-printed polymers is limited. In this study, we evaluated the effects of high humidity and temperature changes on the durability and strength properties of 3D-printed parts. Samples made of commonly available materials such as ABS (Acrylonitrile Butadiene Styrene), ASA (Acrylonitrile-Styrene-Acrylate), HIPS (High-Impact Polystyrene), and PLA (Poly(lactic acid)) were subjected to temperature cycling, from an ambient temperature to −20 °C, and then were heated to 70 °C. After thermal treatment, the samples were subjected to cyclic loading to determine changes in their fatigue life relative to non-thermally treated reference samples. The results of cyclic testing showed a decrease in durability for samples made of ASA and HIPS. The ABS material proved to be resistant to the environmental effects of shocks, while the PLA material exhibited an increase in durability. Changes in the internal structure and porosity of the specimens under temperature changes were also evaluated using microcomputed tomography (microCT). Temperature changes also affected the porosity of the samples, which varied depending on the material used.

## 1. Introduction

Fused filament deposition modeling is one of the most widely used rapid prototyping technologies. Using tools compatible with computer-aided design, this technology enables two embeddings of geometrically complex functional and practical parts. One of the most widespread 3D printing processes is based on the Fused Deposition Modeling (FDM) method. [1,2]. It enables the 3D printing of three-dimensional objects by depositing a solid and continuous working material that is laid down from the base in a layer-by-layer strategy, starting from the bottom and building up to the top. In contrast, this technology is based on hardening liquid resin using a laser beam reflected by a mirror [3]. Wide-ranging studies related to the analysis of the influence of various process parameters on the mechanical properties of parts produced by 3D printing have already been conducted. These previous studies have mainly focused on the influence of manufacturing variables on the strength of materials, namely the influence of filling [4], printing temperature and speed [5], the orientation of the raster along with the air gap [6], printing speed, the size of the nozzle, and the thickness of the layer on the walls of the printout [7]. The results clearly show the correlation between the manufacturing parameters and the quality and durability of the materials. Other studies have addressed the influence of the working environment on material properties, allowing for a better identification of potential applications and uses for prints with different degrees of complexity. The information obtained from these experiments also provided valuable knowledge on the influence of various factors on the quality, durability, and mechanical properties of these products. This enables engineers and home users to select more efficient and durable materials, which translates into a higher quality and durability of manufactured parts.

Review papers that address issues related to assessing the impact of environmental factors on materials used in 3D printing are a valuable source of knowledge [8,9,10]. Based on this information, four 3D-printed materials were chosen for the experiments: ABS, ASA, HIPS, and PLA. They were selected for this study because they are commonly used, due to their easy availability, low price, and unique properties, which make them adequate for distinct applications. Three materials are from the styrene group: ABS (Acrylonitrile Butadiene Styrene), ASA (Acrylonitrile-Styrene-Acrylate), and HIPS (High-Impact Polystyrene). These are materials synthesized from petroleum products. The former is a plastic with high impact strength, elasticity, and good resistance to high temperatures and UV radiation. Its applications include, for example, automotive parts and tools. The second, ASA, is distinguished by its resistance to environmental conditions, which allows for the preparation of dedicated components for outdoor structures. HIPS is used in the printing of large models and is used as a support in a combination of the previously mentioned materials. It is used in the production of various interior applications, such as food packaging and refrigerator liners. The fourth material, PLA (Poly(lactic acid)), belongs to the biodegradable group. As a polymer of biological origin, it is produced from corn starch, which allows it to be used in contact with food. It is very often used to create decorative models [10,11,12,13]. 

These materials are often subjected to the effects of increased humidity and decreases and increases in temperature, concerning ambient temperature, i.e., so-called thermal shocks. Such conditions can occur in the surroundings of a component during its operation and significantly affect its mechanical properties. This article expands on a previous publication that covered the evaluation of changes in strength and chemical properties caused by different shock cycle histories [14]. Other authors have also addressed the impact of distinct potentially degrading factors on material responses, with each examined individually, such as the impact of seawater [15,16], high temperatures [17,18], and sub-zero temperatures [19,20]. The results of these studies showed an increase in strength only when exposed to high temperatures. In contrast, few publications have evaluated the combined effect of potentially adverse factors on the material strength and fatigue life of 3D-printed materials. In this study, we performed low-cycle fatigue tests in polymeric materials for 3D printing that were subjected to environmental degradation. So far, there are very few publications in the literature that evaluate the durability of 3D printing materials through cyclic testing. The authors of [21] studied the effect of the degree of PLA filling on low-cycle fatigue and concluded that the best durability can be achieved by maximizing the filling of the samples. Müller et al. [22] compared the low-cycle fatigue properties of pure 3D-printed PLA and 3D-printed PLA reinforced with pine, bamboo, and cork. The tests showed no significant effect on tensile strength and associated durability. The impact of environmental factors on material durability was also discussed by our team in a recent article focused on changes in the low-cycle fatigue resistance of 3D-printed polymers due to mineral oil and elevated temperatures [23].

The purpose of this study is to determine how environmental conditions, in the form of the exposure of samples to moisture followed by thermal shock cycles, affect the durability of 3D-printed materials. Such an assessment will allow for a more precise selection of materials for specific applications. This, in turn, can lead to the increased efficiency, durability, and safety of products and structures printed by additive manufacturing. This study determined the cyclic strength by measuring the number of loading cycles until failure and evaluated the changes in porosity to correlate the effects of environmental conditions, such as thawing and freezing, with the mechanical properties of 3D-printed parts.

## 2. Materials and Methods

### 2.1. Manufacturing Details

**Figure 1:**
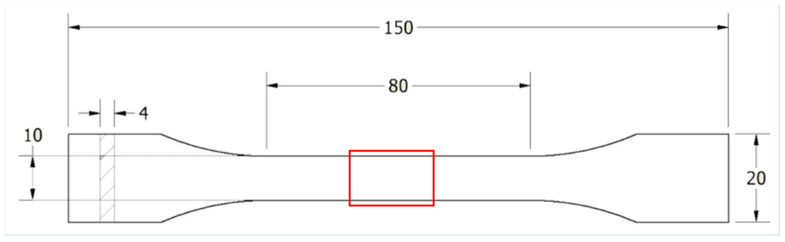
Shape and dimensions of the sample used for testing. Prepared according to EN ISO 527-1:2012 standards [25].

The materials used in the experiments are commercially available from Spectrum (Spectrum Filaments Group, Pęcice Małe, Poland). The full names of the materials used are smart ABS, HIPS-X, ASA 275, and PLA Premium [24]. A sample with detailed dimensions is shown in Figure 1. The fabrication of the samples was executed utilizing a Zortrax m200 plus printer (Zortrax S.A., Olsztyn, Poland). The parameters for the printing process were established based on the default recommendations provided by the manufacturer in ZSuit software (v 2.32.0.0). We have described the details of the parameters used in this paper [14]. This software, supplied with a printer, offers a multitude of adjustable parameters for the printing process tailored to each material, including nozzle temperature, printing speed, layer thickness, and layer height, among others. In the context of our experiment, the only deviation from the default parameters was the adjustment of the fill to 100% and the selection of a linear pattern. This modification resulted from the sample fabrication methodology developed in previous research [14]. This approach ensures the reproducibility and consistency of the sample production process, thereby enhancing the reliability of our experimental results. 

### 2.2. Thermal Shock Cycles

The samples made of ABS, ASA, HIPS, and PLA were placed in water for 72 h. Then, after being removed from the water, the samples were placed in a freezer, where the temperature was −20 °C, for 24 h. After removal from the freezer, the samples in separate batches were transferred to a 70 °C dryer for 72 h. The described cycle was called ‘shock’. Samples from the mentioned materials were divided into three groups: one exposed to 1 shock cycle, another exposed to 2 shock cycles, and another exposed to 3 shock cycles. A reference group (G0) consisting of samples not subjected to thermal conditioning was also considered. 

### 2.3. Static Tests

Static tensile tests were carried out for specimens from the reference group G0 (no subjected thermal cycles) using an Instron E3000 testing machine (Instron, Canton, MA, USA) for each material we tested. The tests were performed following the recommendations outlined in the standard for plastics, EN ISO 527-1:2012 [25]. The strain rate was 1 mm/min. The specimen geometry used in these tests is shown in Figure 1. This is based on the values of maximum stress, σ_uts_, obtained from the experiments. Exemplary graphs on the base, which was set to σ_uts_, are shown in our paper [14].

The load levels in the cyclic tests were determined for the specimens in the other groups. The tensile strength values and load levels for the different polymers are shown in Table 1. The stress levels in cyclic tests were set at values of 90, 80, 70, 60, and 50% of the tensile strength σ_uts_ for the reference group G0. For each material, 5 samples were tested.

### 2.4. Cyclic Tests

An Instron E3000 testing machine was used to evaluate the fatigue strength under low-cycle fatigue conditions. The tests were carried out using load control mode at pulsating loading conditions (R = 0) using sinusoidal cyclic waves. The stress amplitude (σ_a_) was expressed as a percentage of the tensile strength, as described in Section 2.3. For each material, five load levels were studied [14]. On each level of load, 5 samples were tested. The tests were conducted at a frequency of 2 Hz, and the number of loading cycles completed until specimen failure was recorded.

### 2.5. Porosity Measurement

To evaluate the modifications in the internal structure of the samples after exposure to temperature changes, this study was carried out using X-ray microtomography (micro-CT). This study was performed with a viva CT 80, whose manufacturer is Scanco (Scanco. A.G., Brüttisellen, Switzerland). The scanning parameters used included 55 kVp, 145 mA, 0.5 mm Alu filter, 200 ms integration time, and 24 µm resolution. The scanning time for a single sample was 66 min. During the test, the changes in porosity for the samples after each shock cycle were evaluated and correlated with the results from the control group. Each specimen was examined on the middle part at a length of 10 mm. These analyses were carried out before the low-cycle fatigue tests. The region considered in the scanning analysis of each sample is marked in a red rectangle in Figure 1.

### 2.6. Statistics 

Origin 8.6 Pro software was used to analyze the results, which includes built-in modules for statistical analysis. The first step of the analysis was to confirm the normality of the data distribution using the Shapiro–Wilk test and to check the homogeneity of variance using Levene’s test. Then, the ANOVA test (analysis of variance) was conducted, which allowed us to determine the significance of differences between the mean values of the results obtained in individual groups. To compare groups that showed significant differences in results, the Tukey post hoc test was used. All analyses were conducted assuming a significance level of *p* < 0.05. This method is consistent with the approach used in our previous publication. All procedures were carried out using the principles of a scientific approach to statistical analysis [23]. 

## 3. Results

**Figure 2:**
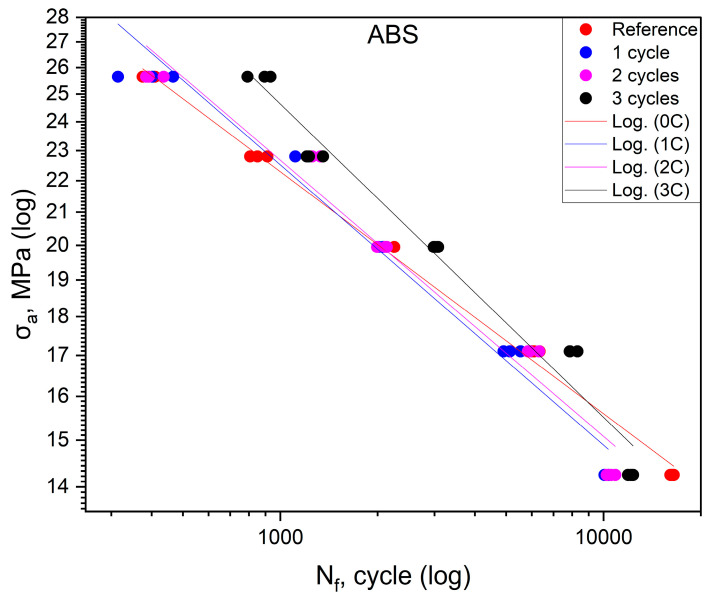
Fatigue curves for ABS.

The fatigue life values obtained in the tests for ASA are summarized in Table 2. The effect of shock cycles on fatigue life for ABS is shown in Figure 2. The fatigue curves of the base material and the materials after one, two, and three shock cycles are also shown in the figure. The position of the curves relative to each other indicates that there is no significant effect on the fatigue life of the material of the first and second shock cycles. Some differences are evident for the material after three shock cycles. An increase in durability is apparent, especially in the range of values of 70–90% of the static tensile strength value. For values of 50%, the durability of the material after three cycles is less than that of the base samples. Based on the position of the curves about each other, it can be concluded that the shock cycles do not affect the deterioration of the fatigue life of the ABS material. After three cycles, an increase in this durability is even visible, especially in the range above 70% of the static tensile strength value. 

**Figure 3:**
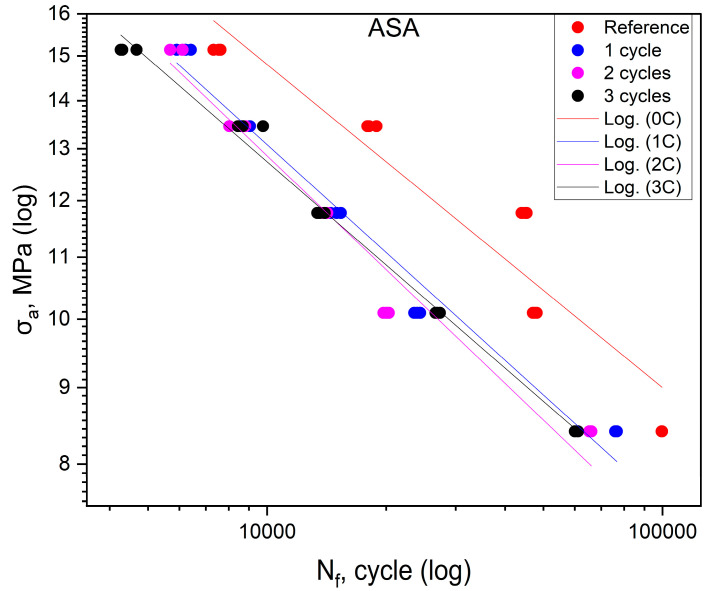
Fatigue curves for ASA.

The fatigue life results obtained in the experiments for ASA are listed in Table 3. Figure 3 shows the effect of shock cycles on fatigue life for ASA. The fatigue curves of the materials after one, two and three cycles are shifted toward the area of lower durability concerning the base material. The position of the curves obtained for the specimens after one, two, and three cycles indicates a clear effect of the shock cycles on the reduction of the fatigue life of ASA. On the other hand, the mutual position of curves that overlap indicates that the number of shock cycles does not significantly affect the reduction in durability. The reduction in durability for the material after one cycle is similar to that of the material after two and three cycles. Since all the S-N curves in the figure are almost parallel, it can be concluded that the fatigue life reduction is similar over the entire range of stress values at which the tests were conducted.

**Figure 4:**
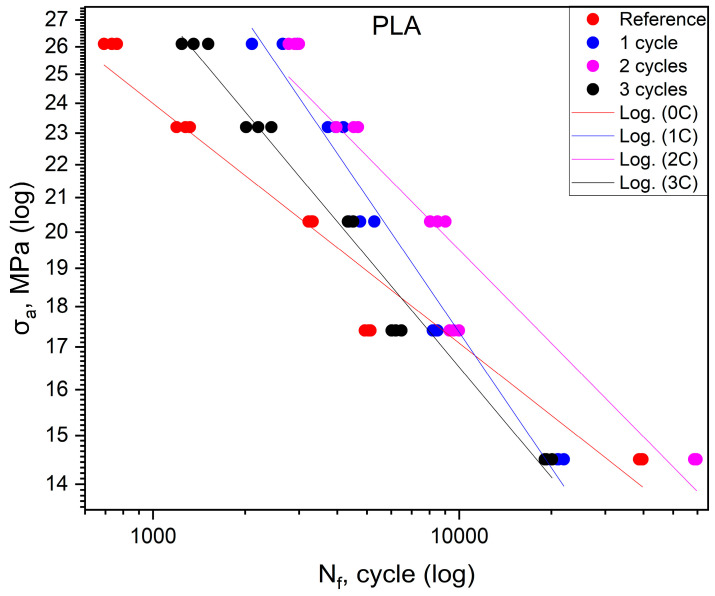
Fatigue curves for PLA.

The fatigue life results obtained for PLA are compiled in Table 4. Figure 4 shows the effect of shock cycles on the fatigue life of PLA. The fatigue curves of the material after one, two, and three shock cycles have different positions. The figure shows that for specimens after one and three cycles at stress values above 17 ÷ 18 MPa, the fatigue life relative to the reference group increased, and, below, this value decreased. The situation is different for samples after two thermal cycles. In this case, the fatigue life increased over the entire range of stresses tested, but a comparison of the slope angle of the curve for this group and that of the control group shows that the increase in fatigue life in this group is greater for higher stress percentages σ_uts_.

**Figure 5:**
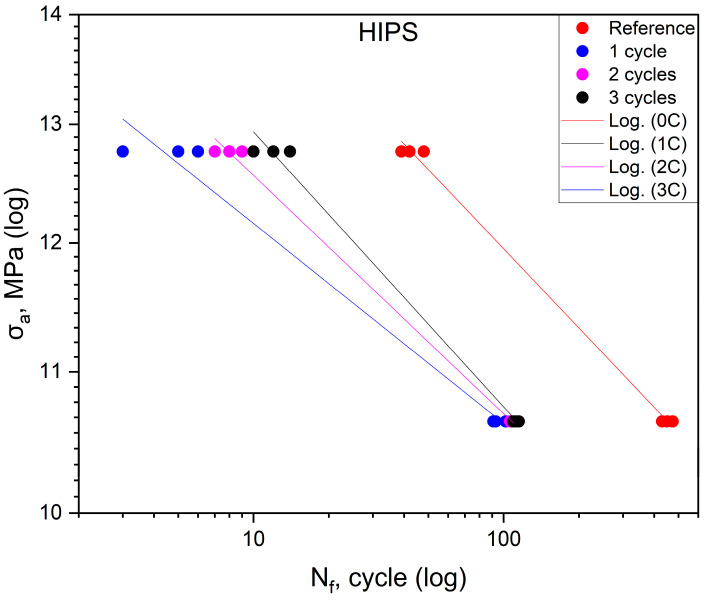
Fatigue curves for HIPS.

Table 3 displays the fatigue life results for HIPS. Figure 5 shows the effect of shocks on the fatigue life of the HIPS polymer. For this material, the tests describe fatigue life only in the range below 60% of the maximum stress value obtained from the static tensile test. The reason for this is that there was a marked decrease in fatigue life values after the first shock cycle. Compared to the value of the maximum stress obtained in the reference group, there is a decrease of more than 30% in fatigue life. This is indicated by the data shown in Table 5. The specimens in the cyclic test failed during the first loading cycle before reaching the target stress at which the test was to be conducted. The stress value at which the specimens failed after shock cycling was in the range of 62–67% of the maximum stress σ_uts_ obtained from the static tensile test of the reference group specimens. Therefore, fatigue plots were created based on only two measurement points, appropriate for stresses equal to 50 and 60% of the σ_uts_ value obtained from the static tensile test of the control group. 

The analysis of Figure 2, Figure 3, Figure 4 and Figure 5, as discussed above, shows different effects of the thermal shock cycles on the S-N curves obtained for the distinct cases and tested materials. These differences may be due to the porosity degree, which is a characteristic of each material, as well as their ability to absorb water during the thermal shock cycles, which is also different. This relationship between porosity, thermal shocks, and fatigue loading leads to complex damage mechanisms. This triangular relationship will be addressed in detail in a follow-up study focused on both chemical alterations and damage micromechanisms.

Table 6 shows the equations of fatigue curves for the graphs shown in Figure 2, Figure 3, Figure 4 and Figure 5. In Figure 2, Figure 3, Figure 4 and Figure 5, taking in log(σ_a_) − log(N_f_) coordinates (σ_a_—stress amplitude, N_f_—the number of loading cycles until failure), the points corresponding to the results obtained from each sample are plotted. These points are described by the regression line and the value of the coefficient of determination. To check whether the regression line well describes the obtained results, a Student’s *t*-test was carried out with an assumed significance level of *p* = 0.05. The results of the *t*-test in all cases showed the correctness of the adopted regression curves. In addition, the normality of the distribution of the residuals was checked using the Shapiro–Wilk test. The results of the test indicated that there were no grounds for questioning the normality of the distribution of the results.

To check whether the results obtained in each group differ from each other, they were described by regression lines. Then, for each material, an equality test for the directional coefficients and free expressions of the regression equations describing them was carried out. The results of the tests confirmed that the regression curves describing the results obtained for each group are statistically different. 

### 3.1. Structure Evaluation Using Microcomputed Tomography (microCT)

#### 3.1.1. Sample Preparation and Testing Process

During the test, changes in the porosity of samples from each group after the first, second, and third thermal cycles were evaluated in comparison with the G0 control group. The comparison was carried out at a length of 10 mm in the middle of the working part of each sample. In each group, for each material, five samples were tested.

#### 3.1.2. Results and Statistical Analysis of Porosity Changes 

**Figure 6:**
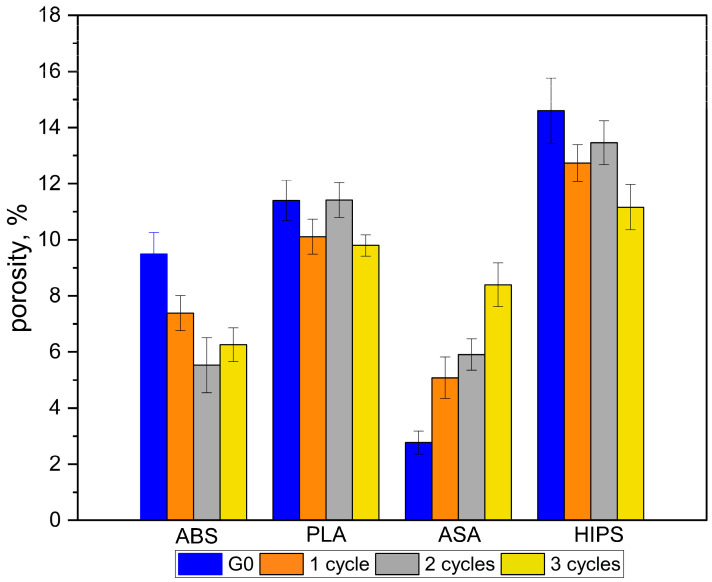
Changes in porosity values for materials after successive thermal shock cycles.

**Figure 7:**
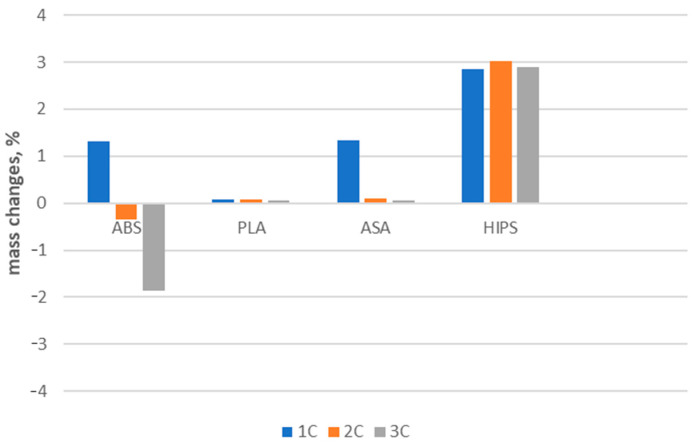
Changes in mass of sample after successive cycles.

The porosity values of the samples are shown in Table 7. It can be seen that, for different materials, the changes have a different course. For ABS, HIPS, and PLA, the porosity of the material decreases after thermal cycling. For ASA, the porosity increases as the number of thermal cycles to which the material has been subjected increases. These trends are shown in Figure 6. In this figure, the height of the bar indicates the average value of porosity with the values of the standard deviation. Figure 7 shows changes in the mass of samples after successive cycles. It is clear that the change in porosity does not correspond with the changed mass of the samples. Further, the parameters are impossible to measure and so demand additional examination.

To confirm the direction of changes in porosity, an analysis was carried out to evaluate the differences in the mean values of porosity in the reference group and after the thermal cycles. The porosity results in all groups were characterized by a normal distribution (checked by the Shapiro–Wilk test, with a significance level of *p* = 0.05), and variance values were also comparable. One-way ANOVA analysis was used, which showed that the mean values of porosity in each group of materials differed statistically. A Tukey post-hoc test was used to identify which groups differed in their mean porosity values. The results of the Tukey test are shown in Table 8. Both the Anova test and the Tukey post-hoc test were conducted at a significance level of *p* = 0.05. 

The results of the statistical analyses indicate significant statistical differences for ABS and HIPS (bolded type in Table 8). For ABS and PLA, the trends seen in Figure 6 could not be statistically confirmed at the accepted significance level of *p* = 0.05. Quantitatively evaluating the porosity changes in individual materials, significant differences can be observed, especially for ABS and ASA. 

**Figure 8:**
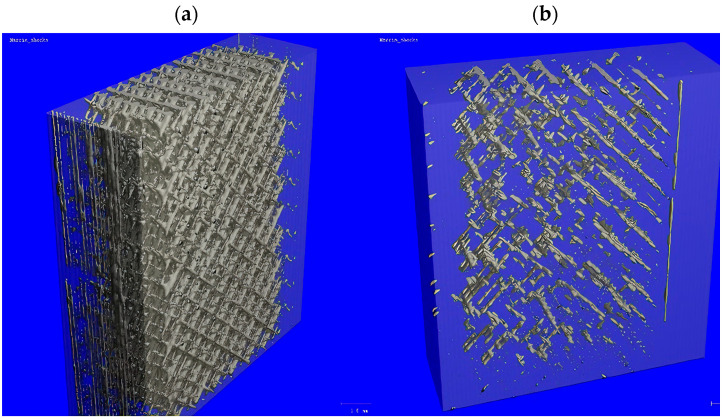
Changes in porosity values for exemplary samples: (**a**) HIPS; (**b**) ASA.

Taking the porosity values in the G0 group for ABS and ASA as a reference, the maximum changes in porosity values are −41.3% for ABS (G0 vs. 2C) and +311.43% for ASA (G0 vs. 3C). For HIPS and PLA, the changes in porosity are less than ±30% compared to the baseline value for the G0 group. It should be pointed out that the noted trends of increase or decrease in porosity were not fully confirmed by the results of statistical tests. Figure 8 shows the distribution of pores in a piece of a sample (dimensions: 4 × 10 × 10 mm) made of HIPS with a porosity of 14.83% (Figure 8a) and of a sample made of ASA with a porosity value of 2.71% (Figure 8b). Detailed analyses of the amount and nature of porosity changes require the examination of a larger number of samples, which is beyond the scope of this work. Due to the complexity of the problem, this will be addressed in a subsequent article.

## 4. Discussion

For specimens subjected to shock exposure from made of ABS material, the position of the fatigue curves (Figure 2) shows that there is no significant effect on fatigue life for the first two cycles, while for the third cycle, an increase in life is apparent in the stress range above 16 MPa. For values below 50% of the ultimate tensile strength, the durability of the material specimens is lower compared to the reference. In the case of ASA (Figure 3), the results of cyclic tensile fatigue tests showed a clear reduction in fatigue life for the material subjected to the first shock cycle. Subsequent cycles did not cause any further decrease in fatigue life. Samples made of PLA material (Figure 4) for the first and third cycles showed an increase in durability compared to the reference group at a stress level of 20.3 MPa. In addition, specimens subjected to two shock cycles showed an increase in durability over the entire load range tested, with the increase being greater the higher the stress value σ_a_. HIPS (Figure 5) proved to be the most sensitive to the effect of thermal cycling. After one shock cycle, there was a decrease in the fatigue strength of the samples by more than 30%. For this reason, performing the test at 90, 80, and 70% was not possible. Testing under cyclic loading was only possible at σ_a_ values lower than 60% of σ_uts_ values. 

In the next stages of research, we intend to carry out analyses regarding the nature of changes in energy absorption, to assess the nature and size of changes in dissipation energy during cyclic tests [26,27,28,29], to evaluate chemical and microstructural changes associated with shock cycle characteristics, and to build fatigue life models sensitive to both loading histories and environmental effects. Recently, Vidinha et al. [30,31] developed an energy-based approach to derive the S-N curves for fiber-reinforced composite materials subjected to both fatigue loading and long-term immersion in seawater. This promising approach only requires a reference S-N curve obtained in the absence of environmental degradation and a numerical model that simulates the degradation caused by the environmental media. This model has been successfully tested for immersion times for up to 910 days. 

The microCT results have no clear interpretation. They showed for both the ABS and HIPS materials that porosity decreased after the application of thermal shocks. For ASA, the porosity increased. However, these trends were not confirmed by the results of statistical analyses. The changes in porosity may have been due to changes in the external dimensions of the samples. During visual inspection, it was found that the samples did not undergo any visible shape deformation after thermal shocks. However, a detailed quantitative analysis of dimensional changes was not performed. For these reasons, changes in porosity require a separate analysis, for which a larger number of specimens need to be examined. Many additional tests need to be performed using techniques used in the study of other porous materials such as foams, ceramics, or trabecular bone [32,33,34,35,36,37].

## 5. Conclusions

The present paper studied the effect of thermal shocks on the low-cycle fatigue behavior of four polymeric materials (ABS, ASA, HIPS, and PLA). Porosity levels associated with the different materials and the number of thermal shock cycles applied to the materials were evaluated via microtomography. The results indicate for the ABS material, the effect of thermal cycling does not affect its fatigue life. For ASA and HIPS materials, a reduction in fatigue life is noticeable. For this reason, based on the results obtained, it is not possible to confirm its usefulness for operation in conditions involving temperature changes and high humidity. In the case of the PLA material, it can be assumed that the changes in fatigue life under the influence of increased humidity and temperature variations are random. Based on the tests carried out in this investigation, it was not possible to unambiguously indicate the trend of changes in durability. Therefore, we believe that for this material, it is not necessary to carry out additional tests to assess whether the nature of the changes occurring would result in an improvement or decrease in durability. Thus, based on the results of our tests, we can conclude that ABS is the most suitable material for parts that will work in an environment with increased humidity and temperature changes. Regarding the wide range of trends observed for the different materials, it is believed that these changes are the result of a triangular relationship between porosity, water absorption, and fatigue loading. An assessment of the chemical alterations and the damage accumulation micromechanisms can help to better understand the specificities of each material. This assessment will be addressed in a follow-up study.

## Figures and Tables

**Table 1:** Tensile strength σ_uts_ values for samples from the reference group G0.

Material	ABS	ASA	PLA	HIPS
σ_uts_ (MPa)	28.5 (0.86)	16.82 (0.31)	29 (0.07)	21.27 (0.37)

(…)—standard deviation.

**Table 2:** Fatigue life values obtained from cyclic test for ABS material.

ABS	Reference		One Cycle	Two Cycles	Three Cycles
	σ_a_ MPa	N_f_, Cycle	N_f_, Cycle	N_f_, Cycle	N_f_, Cycle
90%	25.6	390 ^b^ (14.3)	394 ^b^ (75.8)	404 ^b^ (27.6)	873 ^a^ (73)
80%	22.8	856 ^b^ (43.3)	1265 ^a^ (131.9)	1270 ^a^ (57.4)	1264 ^a^ (80.9)
70%	19.9	2127 ^b^ (97.9)	2031 ^b^ (46.3)	2082 ^b^ (76.3)	3027 ^a^ (47.2)
60%	17.1	6145 ^b^ (149.4)	5193 ^c^ (320.4)	5554 ^b^ (252.1)	8016 ^a^ (253)
50%	14.2	16,309 ^a^ (159.9)	10,193 ^c^ (132.3)	10,557 ^c^ (321.6)	12,139 ^b^ (221.8)

Index indicates homogeneous groups within a single material. Letters (a, b, and c) stand for homogeneous groups.

**Table 3:** Fatigue life values obtained from cyclic test for ASA material.

ASA	Reference		One Cycle	Two Cycles	Three Cycles
	σ_a_, MPa	N_f_, Cycle	N_f_, Cycle	N_f_, Cycle	N_F_, CYCLE
90%	15.1	7628 ^a^ (254.4)	6182 ^b^ (255.7)	5969 ^b^ (244.6)	4417 ^c^ (227.1)
80%	13.5	18,190 ^a^ (565.6)	8802 ^b^ (240.3)	8523 ^b^ (430.4)	8964 ^b^ (320)
70%	11.8	44,750 ^a^ (544)	14,939 ^b^ (451.4)	13,863 ^b,c^ (326.7)	13,658 ^c^ (290)
60%	10	47,531 ^a^ (427.4)	23,977 ^c^ (411)	20,023 ^d^ (307.2)	26,976 ^b^ (340.1)
50%	8.4	99,530 ^a^ (149)	76,306 ^b^ (523.3)	65,493 ^c^ (545.1)	60,542 ^d^ (564.4)

Index indicates homogeneous groups within a single material. Letters (a, b, c, and d) stand for homogeneous groups.

**Table 4:** Fatigue life values obtained from cyclic test for PLA material.

PLA	Reference		One Cycle	Two Cycles	Three Cycles
	σ_a_, MPa	N_f_, Cycle	N_f_, Cycle	N_f_, Cycle	N_f_, Cycle
90%	26.1	729 ^c^ (29.1)	2577 ^a^ (438.6)	2894 ^a^ (111)	1372 ^b^ (137.1)
80%	23.2	1263 ^c^ (52.3)	3956 ^a^ (230)	4392 ^a^ (368.6)	2220 ^b^ (211.3)
70%	20.3	3280 ^c^ (41.6)	4800 ^b^ (461.3)	8518 ^a^ (495.5)	4449 ^b^ (99.8)
60%	10	5038 ^d^ (87.4)	8344 ^b^ (148.1)	9626 ^a^ (343.2)	6234 ^c^ (230.6)
50%	14.5	39,267 ^b^ (420.6)	21,343 ^c^ (558.5)	59,129 ^a^ (546.3)	19,472 ^d^ (556.8)

Index indicates homogeneous groups within a single material. Letters (a, b, c, and d) stand for homogeneous groups.

**Table 5:** Fatigue life values obtained from cyclic test for HIPS material.

HIPS	Reference		One Cycle	Two Cycles	Three Cycles
	σ_a_, MPa	N_f_, Cycle	N_f_, Cycle	N_f_, Cycle	N_f_, Cycle
90%	19.1	-	-	-	-
80%	17	-	-	-	-
70%	14.9	-	-	-	-
60%	12.8	43 ^a^ (3.7)	5 ^c^ (1)	8 ^b,c^ (2)	12 ^b^ (2)
50%	10.6	451 ^a^ (18)	95 ^b^ (9)	107 ^b^ (7)	112 ^b^ (11)

Index indicates homogeneous groups within a single material. Letters (a, b, and c) stand for homogeneous groups.

**Table 6:** Equations of the fatigue curves are shown in Figure 2, Figure 3, Figure 4 and Figure 5.

Group	ABS	ASA	PLA	HIPS
G0	log (N_f_) = −3.01 log (σ_a_) + 43.308 R^2^ = 0.9912	log (N_f_) = −2.61 log (σ_a_) + 38.779 R^2^ = 0.946	log (N_f_) = −2.861 log (σ_a_) + 43.724 R^2^ = 0.9232	log (N_f_) = −1.062 log (σ_a_) + 16.99 R^2^ = 0.9968
1C	log (N_f_) = −3.57 log (σ_a_) + 47.681 R^2^ = 0.9876	log (N_f_) = −2.637 log (σ_a_) + 37.489 R^2^ = 0.9446	log (N_f_) = −5.359 log (σ_a_) + 67.029 R^2^ = 0.9231	log (N_f_) = −1.277 log (σ_a_) + 18.265 R^2^ = 0.9919
2C	log (N_f_) = −3.513 log (σ_a_) + 47.408 R^2^ = 0.9878	log (N_f_) = −2.771 log (σ_a_) + 38.533 R^2^ = 0.93	log (N_f_) = −3.686 log (σ_a_) + 53.891 R^2^ = 0.8672	log (N_f_) = −1.137 log (σ_a_) + 15.956 R^2^ = 0.9969
3C	log (N_f_) = −3.969 log (σ_a_) + 52.171 R^2^ = 0.9881	log (N_f_) = −2.624 log (σ_a_) + 37.087 R^2^ = 0.989	log (N_f_) = −4.398 log (σ_a_) + 57.188 R^2^ = 0.9625	log (N_f_) = −1.631 log (σ_a_) + 17.776 R^2^ = 0.9923

**Table 7:** Average porosity values [in %] for materials after cyclic loading.

Group	ABS	ASA	HIPS	PLA
G0	9.52	2.71	14.83	11.45
1C	7.37	5.01	12.64	10.05
2C	5.55	5.94	13.43	11.45
3C	6.25	8.44	11.19	9.83

**Table 8:** Tukey test results.

		ABS					ASA		
group	G0	1C	2C	3C	group	G0	1C	2C	3C
G0	-	**S**	**S**	**S**	G0	-	NS	S	S
1C	-	-	S	NS	1C	-	-	NS	S
2C	-	-	-	NS	2C	-	-	-	S
		**HIPS**					**PLA**		
group	G0	1C	2C	3C	group	G0	1C	2C	3C
G0	-	S	NS	**S**	G0	-	S	NS	S
1C	-	-	NS	**S**	1C	-	-	S	NS
2C	-	-	-	**S**	2C	-	-	-	S

S—significant; NS—nonsignificant.

## Data Availability

Shock data are contained within the article. The Micro CT data supporting this study’s findings are available from the corresponding authors on request. The Micro CT data are not publicly available because we have not prepared a data package and they will be used in the next article. They will then be published as a larger data package.
